# Análise das Estratégias de Revascularização em Doentes com Infarte Agudo do Miocárdio em Choque Cardiogênico – Resultados do Registro Português de Síndromes Coronárias Agudas

**DOI:** 10.36660/abc.20210127

**Published:** 2021-05-06

**Affiliations:** 1 Universidade Federal do Rio de Janeiro Rio de JaneiroRJ Brasil Universidade Federal do Rio de Janeiro, Rio de Janeiro, RJ – Brasil.; 2 Universidade de Vassouras VassourasRJ Brasil Universidade de Vassouras, Vassouras, RJ – Brasil.; 3 Centro Universitário de Valença VassourasRJ Brasil Centro Universitário de Valença, Valença, RJ – Brasil.

**Keywords:** Infarto do Miocárdio, Revascularização Miocárdica, Choque Cardiogênico, Síndrome Coronariana Aguda

O Registro Português de Síndromes Coronárias Agudas foi elaborado em 2002 pela Sociedade Portuguesa de Cardiologia a partir da constatação da necessidade de um conhecimento mais amplo sobre a abordagem nacional das síndromes coronárias agudas (SCA) e levou à fundação do Centro Nacional para Coleção de Dados em Cardiologia (CNCDC) para centralizar toda a informação e apoiar a sua análise, o que culminou com a criação dos seus primeiros registros: o Registo Nacional de Síndromes Coronárias Agudas (RNSCA), simultaneamente com o Registo Nacional de Cardiologia de Intervenção. O RNSCA é um registo observacional prospetivo e contínuo. Foram convidados a participar voluntariamente todos os serviços e departamentos de Cardiologia dos hospitais portugueses. A inclusão de doentes teve início em 1 de janeiro de 2002 e mantém-se sem interrupções até o presente momento. Brevemente, foi solicitado a cada centro que incluísse de forma consecutiva doentes internados com o diagnóstico de SCA (inclusive infarto agudo do miocárdio com elevação de ST, sem elevação de ST e angina instável), com base na clínica, no eletrocardiograma e nos biomarcadores de necrose miocárdica. Os dados recolhidos incluem dados demográficos, características basais, evolução laboratorial, evolução clínica, terapêutica efetuada, dados da intervenção percutânea, dados da alta e do seguimento aos seis meses (na primeira fase do registo) ou ao primeiro ano (na segunda fase do registo). Inicialmente, os dados eram preenchidos em papel, posteriormente transferidos para uma base eletrônica e desde 2004 são submetidos diretamente por via eletrônica. Em consulta ao *site* do RNSCA em fevereiro de 2021, este contava com 63.029 registros.^1^^,^^2^

Uma pergunta está presente na abordagem da revascularização da síndrome coronariana aguda que evolui com choque cardiogênico: devemos abordar apenas o vaso culpado ou todas as lesões coronarianas significativas? O estudo das estratégias de revascularização em doentes com infarte agudo do miocárdio em choque cardiogênico, proveniente dos resultados do Registro Português de Síndromes Coronárias Agudas, traz esclarecimentos e ajuda na tomada de decisão.^3^ Atualmente, as recomendações da Sociedade Brasileira de Hemodinâmica e Cardiologia Intervencionista são as seguintes: “A estratégia invasiva buscando a revascularização miocárdica do vaso culpado e, potencialmente, de vasos não culpados com doença coronária significativa, é recomendada nos casos de IAMCSST evoluindo com falência cardíaca e choque cardiogênico irrespectivamente do tempo de apresentação.”^4^

Um estudo multicêntrico brasileiro mostrou que a estratégia de revascularização completa esteve associada à redução significativa dos desfechos primários (óbito cardiovascular, reinfarto ou angina recorrente) e secundários (acidente vascular encefálico, parada cardiorrespiratória não fatal, maior sangramento ou necessidade de nova intervenção) no seguimento de um ano quando comparada à estratégia de revascularização incompleta.^5^

O estudo baseado no RNSCA português envolve pacientes com marcadores prognósticos sabidamente ruins, anatomicamente multiarteriais^6^ e hemodinamicamente em estado de choque cardiogênico,^7^ o que justifica a alta mortalidade relatada no estudo. Os resultados não demonstraram diferença entre a revascularização completa no procedimento-índice *versus* um grupo composto pela revascularização completa diferida ou incompleta, quando relacionados ao desfecho primário composto por morte intra-hospitalar ou reinfarto.^3^ Vale ressaltar que o tempo de seguimento foi mais curto quando comparado ao de estudos semelhantes^8^^–^^10^ para avaliar a estratégia de revascularização na síndrome coronariana aguda e que levavam em conta apenas a internação durante o evento-índice. Os resultados de estudos semelhantes mostraram que a revascularização completa é superior; no entanto, não envolviam apenas pacientes em choque cardiogênico.^5^^,^^8^^–^^10^

Uma grande vantagem demonstrada no estudo3 é a disponibilidade de um registro nacional contínuo, de longa duração, com grande abrangência e múltiplas variáveis que possibilite a realização de diferentes estudos, seguimento temporal de eventos, recomendações ou intervenções e tendência nos números e desfechos das síndromes coronarianas agudas em Portugal. No Brasil, não dispomos de registro semelhante; ele não é impossível de ser feito, mas um país continental, com dois sistemas de saúde distintos, se torna, no mínimo, difícil.
