# Supplementary material for: Análise das Estratégias de Revascularização em Doentes com Infarte Agudo do Miocárdio em Choque Cardiogênico – Resultados do Registro Português de Síndromes Coronárias Agudas
Source: Arq Bras Cardiol. 2021 May 6;116(5):877–8. [Article in Portuguese] doi: 10.36660/abc.20210127 (PMC8121471; doi:10.36660/abc.20210127)
Supplement: Translation

# Translation: Analysis of Revascularization Strategies in Patients with Acute Myocardial Infarction and Cardiogenic Shock - Results from the Portuguese Registry on Acute Coronary Syndromes

**Affiliations:** 1 Rio de JaneiroRJ Brazil Universidade Federal do Rio de Janeiro, Rio de Janeiro, RJ – Brazil.; 2 VassourasRJ Brazil Universidade de Vassouras, Vassouras, RJ – Brazil.; 3 VassourasRJ Brazil Centro Universitário de Valença, Valença, RJ – Brazil.

**Keywords:** Myocardial Infarction, Myocardial Revascularization, Shock, Cardiogenic, Acute Coronary Syndrome

The Portuguese Registry on Acute Coronary Syndromes was drafted in 2002 by the Portuguese Cardiology Society due to the need for a broader knowledge concerning the national approach to Acute Coronary Syndromes (ACS) and created the National Cardiology Data Collection Center (NCDCC) to centralize all of the information and support its analysis, culminating in the creation of its first records: The National Registry of Acute Coronary Syndromes (NRACS) was created simultaneously with the National Register of Interventional Cardiology. The NRACS is a prospective and continuous observational registry. All Portuguese hospital cardiology services and departments were invited to participate voluntarily in this study. The inclusion of patients began on January 1, 2002, and has been maintained to the present day without interruption. Briefly, each center received a request to consecutively include all patients hospitalized with a diagnosis of ACS (including acute ST-elevation myocardial infarction (STEMI), without unstable ST-elevation or angina), based on clinical evaluation, on the electrocardiogram, and on the biomarkers of myocardial necrosis. The collected data include demographic data, baseline characteristics, laboratory evolution, clinical evolution, executed therapy, data on percutaneous intervention, as well as data on hospital discharge and follow-up for six months (in the first stage of the registry) or for one year (in the second stage of the registry). Initially, the data were collected on paper and then transferred to an electronic data base, but since 2004, they are being submitted directly in electronic form. Upon consulting the NRACS e-mail address in February 2021, 62,029 records were found.^1^^,^^2^

One question arises in the ACS revascularization approach, which evolves with cardiogenic shock: Treat only the culprit blood vessel or treat all of the significant coronary lesions? The study of revascularization strategies in patients with acute myocardial infarction in cardiogenic shock stemming from the results of the Portuguese Registry of Acute Coronary Syndromes sheds light on the issue and aids professionals in decision-making.^3^ Currently, the recommendations from the Brazilian Society of Hemodynamics and Interventional Cardiology are as follows: “The invasive strategy seeking to achieve the myocardial revascularization of the culprit blood vessel and, potentially, of the non-culprit blood vessels with significant coronary disease, is recommended in the cases of acute STEMI (IAMCSST) evolving with heart failure and cardiogenic shock, regardless of the time elapsed since its onset.”^4^

One Brazilian multicenter study demonstrated that the complete revascularization strategy was associated with a significant reduction in the primary (cardiovascular death, re-infarction, and recurring angina) and secondary (stroke, non-fatal cardiorespiratory failure, greater bleeding, or the need for reintervention) outcomes in the one-year follow-up when compared to the incomplete revascularization strategy.^5^

The study based on the Portuguese NRACS involves patients with knowingly poor prognostic markers, as they are anatomically multiarterial^6^ and hemodynamically in cardiogenic shock,^7^ which justified the high mortality in the study. The results showed no difference between complete revascularization in the procedure index as compared to a group consisting of differed complete or incomplete revascularization in relation to the primary outcome of intra-hospital death or re-infarction.^3^ It is important to note that the follow-up time was shorter when compared to similar studies^8^^–^^10^ to evaluate the revascularization strategy in ACS, bearing in mind only the hospitalization during the main event. The results of similar studies demonstrate that complete revascularization is superior, but it did not involve only patients in cardiogenic shock.^5^^,^^8^^–^^10^

One major advantage illustrated in this study3 is the availability of a continuous, long-standing, national Registry, with a broad coverage and multiple variables that make it possible to conduct different studies; the temporary follow-up of events, recommendations, or interventions; and the tendency in the numbers and outcomes of ACS throughout Portugal. Brazil lacks a similar registry. It is not impossible to create, but, as a continental country with two different health systems, it is, to say the least, quite difficult.
